# Supplementary material for: A fault-intrusion-tolerant system and deadline-aware algorithm for scheduling scientific workflow in the cloud
Source: PeerJ Comput Sci. 2021 Nov 2;7:e747. doi: 10.7717/peerj-cs.747 (PMC8576554; doi:10.7717/peerj-cs.747)
Supplement: Supplemental Information 6 [file peerj-cs-07-747-s006.zip › Myproject1.1/docs/org/cloudbus/cloudsim/power/PowerVmAllocationPolicyMigrationMedianAbsoluteDeviation.html]

PowerVmAllocationPolicyMigrationMedianAbsoluteDeviation


JavaScript is disabled on your browser.


- Overview
- Package
- Class
- Use
- Tree
- Deprecated
- Index
- Help

- Prev Class
- Next Class

- Frames
- No Frames

- All Classes

- Summary:
- Nested |
- Field |
- Constr |
- Method

- Detail:
- Field |
- Constr |
- Method


org.cloudbus.cloudsim.power

## Class PowerVmAllocationPolicyMigrationMedianAbsoluteDeviation

- java.lang.Object
- - org.cloudbus.cloudsim.VmAllocationPolicy
  - - org.cloudbus.cloudsim.power.PowerVmAllocationPolicyAbstract
    - - org.cloudbus.cloudsim.power.PowerVmAllocationPolicyMigrationAbstract
      - - org.cloudbus.cloudsim.power.PowerVmAllocationPolicyMigrationMedianAbsoluteDeviation

- ---

    

  ```
  public class PowerVmAllocationPolicyMigrationMedianAbsoluteDeviation
  extends PowerVmAllocationPolicyMigrationAbstract
  ```

  The Median Absolute Deviation (MAD) VM allocation policy.
  If you are using any algorithms, policies or workload included in the power package, please cite
  the following paper:
  Anton Beloglazov, and Rajkumar Buyya, "Optimal Online Deterministic Algorithms and Adaptive
  Heuristics for Energy and Performance Efficient Dynamic Consolidation of Virtual Machines in
  Cloud Data Centers", Concurrency and Computation: Practice and Experience (CCPE), Volume 24,
  Issue 13, Pages: 1397-1420, John Wiley & Sons, Ltd, New York, USA, 2012

  Since:
  :   CloudSim Toolkit 3.0

- - ### Constructor Summary

    Constructors

    Constructor and Description

    `PowerVmAllocationPolicyMigrationMedianAbsoluteDeviation(java.util.List<? extends Host> hostList,
    PowerVmSelectionPolicy vmSelectionPolicy,
    double safetyParameter,
    PowerVmAllocationPolicyMigrationAbstract fallbackVmAllocationPolicy)`

    Instantiates a new power vm allocation policy migration mad.

    `PowerVmAllocationPolicyMigrationMedianAbsoluteDeviation(java.util.List<? extends Host> hostList,
    PowerVmSelectionPolicy vmSelectionPolicy,
    double safetyParameter,
    PowerVmAllocationPolicyMigrationAbstract fallbackVmAllocationPolicy,
    double utilizationThreshold)`

    Instantiates a new power vm allocation policy migration mad.
  - ### Method Summary

    Methods

    | Modifier and Type | Method and Description |
    | --- | --- |
    | `PowerVmAllocationPolicyMigrationAbstract` | `getFallbackVmAllocationPolicy()` Gets the fallback vm allocation policy. |
    | `protected double` | `getHostUtilizationMad(PowerHostUtilizationHistory host)` Gets the host utilization mad. |
    | `protected double` | `getSafetyParameter()` Gets the safety parameter. |
    | `protected boolean` | `isHostOverUtilized(PowerHost host)` Checks if is host over utilized. |
    | `void` | `setFallbackVmAllocationPolicy(PowerVmAllocationPolicyMigrationAbstract fallbackVmAllocationPolicy)` Sets the fallback vm allocation policy. |
    | `protected void` | `setSafetyParameter(double safetyParameter)` Sets the safety parameter. |

    - ### Methods inherited from class org.cloudbus.cloudsim.power.PowerVmAllocationPolicyMigrationAbstract

      `addHistoryEntry, areAllVmsMigratingOutOrAnyVmMigratingIn, extractHostListFromMigrationMap, findHostForVm, findHostForVm, getExecutionTimeHistoryHostSelection, getExecutionTimeHistoryTotal, getExecutionTimeHistoryVmReallocation, getExecutionTimeHistoryVmSelection, getMaxUtilizationAfterAllocation, getMetricHistory, getMigrationMapFromUnderUtilizedHosts, getNewVmPlacement, getNewVmPlacementFromUnderUtilizedHost, getOverUtilizedHosts, getPowerAfterAllocation, getSavedAllocation, getSwitchedOffHosts, getTimeHistory, getUnderUtilizedHost, getUtilizationHistory, getUtilizationOfCpuMips, getVmSelectionPolicy, getVmsToMigrateFromHosts, getVmsToMigrateFromUnderUtilizedHost, isHostOverUtilizedAfterAllocation, optimizeAllocation, printOverUtilizedHosts, restoreAllocation, saveAllocation, setVmSelectionPolicy`
    - ### Methods inherited from class org.cloudbus.cloudsim.power.PowerVmAllocationPolicyAbstract

      `allocateHostForVm, allocateHostForVm, deallocateHostForVm, getHost, getHost, getVmTable`
    - ### Methods inherited from class org.cloudbus.cloudsim.VmAllocationPolicy

      `getHostList, setHostList`
    - ### Methods inherited from class java.lang.Object

      `clone, equals, finalize, getClass, hashCode, notify, notifyAll, toString, wait, wait, wait`

- - ### Constructor Detail


    - #### PowerVmAllocationPolicyMigrationMedianAbsoluteDeviation

      ```
      public PowerVmAllocationPolicyMigrationMedianAbsoluteDeviation(java.util.List<? extends Host> hostList,
                                                             PowerVmSelectionPolicy vmSelectionPolicy,
                                                             double safetyParameter,
                                                             PowerVmAllocationPolicyMigrationAbstract fallbackVmAllocationPolicy,
                                                             double utilizationThreshold)
      ```

      Instantiates a new power vm allocation policy migration mad.

      Parameters:
      :   `hostList` - the host list
      :   `vmSelectionPolicy` - the vm selection policy
      :   `safetyParameter` - the safety parameter
      :   `utilizationThreshold` - the utilization threshold


    - #### PowerVmAllocationPolicyMigrationMedianAbsoluteDeviation

      ```
      public PowerVmAllocationPolicyMigrationMedianAbsoluteDeviation(java.util.List<? extends Host> hostList,
                                                             PowerVmSelectionPolicy vmSelectionPolicy,
                                                             double safetyParameter,
                                                             PowerVmAllocationPolicyMigrationAbstract fallbackVmAllocationPolicy)
      ```

      Instantiates a new power vm allocation policy migration mad.

      Parameters:
      :   `hostList` - the host list
      :   `vmSelectionPolicy` - the vm selection policy
      :   `safetyParameter` - the safety parameter
  - ### Method Detail


    - #### isHostOverUtilized

      ```
      protected boolean isHostOverUtilized(PowerHost host)
      ```

      Checks if is host over utilized.

      **Specified by:**
      :   `isHostOverUtilized` in class `PowerVmAllocationPolicyMigrationAbstract`

      Parameters:
      :   `_host` - the \_host

      Returns:
      :   true, if is host over utilized


    - #### getHostUtilizationMad

      ```
      protected double getHostUtilizationMad(PowerHostUtilizationHistory host)
                                      throws java.lang.IllegalArgumentException
      ```

      Gets the host utilization mad.

      Parameters:
      :   `host` - the host

      Returns:
      :   the host utilization mad

      Throws:
      :   `java.lang.IllegalArgumentException`


    - #### setSafetyParameter

      ```
      protected void setSafetyParameter(double safetyParameter)
      ```

      Sets the safety parameter.

      Parameters:
      :   `safetyParameter` - the new safety parameter


    - #### getSafetyParameter

      ```
      protected double getSafetyParameter()
      ```

      Gets the safety parameter.

      Returns:
      :   the safety parameter


    - #### setFallbackVmAllocationPolicy

      ```
      public void setFallbackVmAllocationPolicy(PowerVmAllocationPolicyMigrationAbstract fallbackVmAllocationPolicy)
      ```

      Sets the fallback vm allocation policy.

      Parameters:
      :   `fallbackVmAllocationPolicy` - the new fallback vm allocation policy


    - #### getFallbackVmAllocationPolicy

      ```
      public PowerVmAllocationPolicyMigrationAbstract getFallbackVmAllocationPolicy()
      ```

      Gets the fallback vm allocation policy.

      Returns:
      :   the fallback vm allocation policy


- Overview
- Package
- Class
- Use
- Tree
- Deprecated
- Index
- Help

- Prev Class
- Next Class

- Frames
- No Frames

- All Classes

- Summary:
- Nested |
- Field |
- Constr |
- Method

- Detail:
- Field |
- Constr |
- Method
